# Interpreting the Estimand Framework From a Causal Inference Perspective

**DOI:** 10.2196/88813

**Published:** 2026-05-22

**Authors:** Jinghong Zeng

**Affiliations:** 1 Department of Statistics, University of Auckland, 38 Princes Street, Auckland, 1010, New Zealand, 86 15992428924; 2 Department of Statistics and Programming, Jiangsu Hengrui Medicine (China), Guangzhou, Guangdong, China

**Keywords:** causal inference, clinical trial, estimand, intercurrent event, treatment effect

## Abstract

The International Council for Harmonisation of Technical Requirements for Pharmaceuticals for Human Use published the estimand framework in 2019. The estimand framework aims to clearly define a treatment effect for a clinical question through construction of estimands, and it has been widely applied in clinical trials in the pharmaceutical industry. The estimand framework proposes 5 attributes for an estimand: treatments, variables, target populations, population-level summaries, and intercurrent events. It also proposes the treatment policy strategy, the hypothetical strategy, the composite variable strategy, the while on treatment strategy, and the principal stratum strategy to handle intercurrent events. When people give clear definitions for these 5 attributes, they clearly define an estimand that represents a treatment effect. From a statistical perspective, a genuine or causal treatment effect is defined through a causal inference framework. This article aims to interpret the estimand framework using a causal inference framework and help researchers understand the differences between estimands and causal treatment effects. From a causal inference framework based on potential outcomes, an individual treatment effect (ITE) is defined by comparison of individual potential outcomes with experimental or control treatments, and the average treatment effect (ATE) of the experimental treatment versus the control treatment is defined as an average of all ITEs. The statistical presentation of the ATE is not equivalent to an estimand. It has the same treatments, variables, target populations, and population-level summaries as an estimand, but intercurrent events are not part of it. Intercurrent events modify the statistical presentation of the ATE through treatments, variables, and target populations, whose impact can be controlled by intercurrent event strategies. I propose that the estimand attributes can be mapped onto the statistical presentation of the ATE, and that intercurrent events act as mediation mechanisms in the attribute mapping process, which provides a novel way to incorporate the causal inference framework into the estimand framework. If the estimand framework is combined with a causal inference framework, it will gain a stronger theoretical foundation. The interpretation of the estimand framework from a causal inference perspective is useful for both industrial and academic clinical trials. Observational studies may also find useful information on causal inference theories in this article.

## Introduction

The International Council for Harmonisation of Technical Requirements for Pharmaceuticals for Human Use (ICH) is an international association that brings regulatory authorities and the pharmaceutical industry together to discuss the scientific and technical aspects of pharmaceutical development and develop international standardized technical guidelines that are implemented by both regulators and pharmaceutical companies [1]. It was originally founded in 1990 and restructured as a nonprofit legal entity in 2015, with regulatory and industry members such as the US Food and Drug Administration [2]. Over the past 40 years, the ICH has proposed more than 70 technical guidelines that cover different aspects of pharmaceutical development, from good clinical practice to data management and statistical analysis [2]. These guidelines provide clear, standardized requirements on datasets, statistical analysis results, and other materials, which facilitates communication between regulators and pharmaceutical companies. Companies should follow ICH guidelines when submitting drug applications to regulators. The most important guideline for statistical analysis is the Statistical Principles For Clinical Trials E9 [3]. E9 was finalized in 1998 and has become an established international statistical principle for clinical trials. In 2017, the ICH drafted the guideline E9 (R1) as an addendum to E9, and published E9 (R1) in 2019 [4]. A core advancement in E9 (R1) is the estimand framework. This framework consists of definitions of estimands and relevant terms, as well as analysis strategies. It aims to improve precision in defining a treatment effect for a clinical question. Since its release, the estimand framework has gained increasing attention in the pharmaceutical industry and academia [5-9]. Many clinical trials have used the estimand framework to develop new drugs for both oncological and nononcological diseases, and professional working groups such as the Oncology Estimand Working Group have been initiated to study how the estimand framework should be better incorporated into pharmaceutical research [10].

In the world of statistics, causal inference is used to define and estimate a genuine treatment effect [11-20]. One widely used causal inference framework is the potential outcome framework. Potential outcomes are what would happen if a participant took a control treatment or instead took another experimental treatment. Potential outcomes are not yet observed. The treatment effect on this participant is defined as some form of difference between the outcomes of the two hypothetical treatment conditions, which compares two treatments or treatment regimens. Then, the individual treatment effect can be extended to the entire population of interest. Both the estimand framework and the the causal inference framework aim to define a treatment effect. I find that the estimand framework itself does not define a treatment effect as well as it intends to. There are many important issues related to causal inference, including unmeasured confounding, noncompliance, and mediation. The estimand framework does not address these problems. It is more like a framework that makes the analysis objectives clearer. What are the relationships and differences between the estimand framework and the causal inference framework? This is my main question in this article. Drury et al [8] also studied this question, but my reasoning is quite different. Drury et al [8] introduced the potential outcome framework and a statistical formula for the estimand and then focused on defining estimands from the estimand framework and the causal inference framework in specific clinical trial examples, with the aim of discussing how the two frameworks are linked. They argued that the two frameworks do not compete. I strongly agree with this. However, they did not explain how the statistical formula for the estimand is developed in the potential outcome framework and how this formula is related to practical statistical analysis. They also did not discuss in detail how intercurrent events and different strategies would affect the causal interpretation, as intercurrent events are a major part of the estimand framework. From my point of view, Drury et al [8] have not yet grasped the nature of the connections and differences. In this article, I will describe in detail how a causal inference framework can be developed based on potential outcomes and how the statistical presentation of a genuine treatment effect can be developed and related to statistical models people actually work with. The causal inference framework comes from my recent paper in *Statistics in Medicine* [11]. It can deal with several important problems in causal inference, such as unmeasured confounding and noncompliance. It is a practical, useful solution for more complex situations. I will also compare the attributes of the estimand with the statistical formula for a treatment effect, and I will discuss in detail how different intercurrent event strategies affect the causal interpretation of a treatment effect. Finally, I will propose a novel way to incorporate the causal inference framework into the estimand framework through attribute mapping.

I would like to explain a little why causal interpretation is needed by discussing two concepts, the intention-to-treat (ITT) principle and clinical significance. First, the estimand framework heavily relies on the ITT principle, or at least, clinical implementations heavily rely on the ITT principle [21-29]. The E9 states that the ITT principle “asserts that the effect of a treatment policy can be best assessed by evaluating on the basis of the intention to treat a subject (ie, the planned treatment regimen) rather than the actual treatment given. It has the consequence that subjects allocated to a treatment group should be followed up, assessed and analysed as members of that group irrespective of their compliance to the planned course of treatment” [3]. The ITT principle is the gold standard in clinical trials, and it usually relies on the benefits of randomization. Randomization creates balance between treatment groups and helps reduce bias from unmeasured confounding and noncompliance in estimation of treatment effects. Here, I discuss unmeasured confounding. Confounders are factors that affect both treatments and endpoints. For example, in a clinical trial that studies whether a new drug can reduce blood pressure, older participants may absorb the drug with more difficulty and already have higher blood pressure. Hence, age affects both the new drug and blood pressure, and thus age is a confounder. If confounders are known and measured in a study, then they are “measured confounders.” If they are unknown to the researchers, or known but not measured in a study, then they are “unmeasured confounders.” In the previous example, if age is not collected in the trial, then age is an unmeasured confounder. Unmeasured confounding is an important source of bias in estimation of a causal treatment effect [11,30-33]. If it is not adjusted for in statistical analysis, treatment effect estimation can be biased. In the previous example, if age is not adjusted for in statistical analysis, the new drug effect is likely to be underestimated because it is likely to lead to smaller decreases in blood pressure among older people. Randomization, due to its nature, is not confounded with treatments and endpoints. Hence, even if unmeasured confounders exist between treatments and endpoints, the ITT principle uses randomization to adjust for it, where estimation of the treatment effect will not be affected by unmeasured confounding. However, under the ITT principle, what is estimated in statistical analysis is not the genuine effect of the treatment of interest, but the effect of the random assignment. If a significant effect can be detected under the ITT principle, it means that the treatment effect of interest is also significant, because randomization affects endpoints only through treatments of interest; however, the genuine magnitude of the treatment effect will not be known.

Second, I distinguish two concepts: statistical significance and clinical significance [34-42]. Statistical significance typically indicates that the differences between groups being compared are significant in statistical hypothesis tests. Clinical significance typically indicates that the differences between groups being compared are significant from a physician’s perspective. The two kinds of significance are different. People rely on statistical significance to provide evidence that a new drug outperforms a placebo or active comparator, while physicians use medical knowledge to judge the actual clinical benefits of a new drug. For example, a new drug might reduce systolic blood pressure by 2 mm Hg compared to a placebo, and this difference might be statistically significant in hypothesis testing, but a physician might still advise that a difference under 5 mm Hg would not sufficiently improve a patient’s conditions, judging that a difference of 2 mm Hg is not clinically significant. In order to better judge clinical significance, it is necessary to more accurately quantify the treatment effect. This idea also works in other areas of pharmaceutical development, such as precision medicine. Knowing the genuine treatment effect helps compare subgroup differences more efficiently. This does not mean that current analysis approaches, such as the ITT principle, are bad. Many effective drugs have been developed with these approaches. However, as technology and medical demands evolve, analysis approaches may also have to evolve to match.

Let me introduce some estimand-related concepts before making comparisons. E9 (R1) includes a glossary , which I’ve provided in Multimedia Appendix 1 [4]. The estimand framework proposes estimands and distinguishes them from estimators and estimates with regard to statistical roles in the estimation of treatment effects. An estimand is a precise definition of a treatment effect in a clinical question. An estimator is a statistical method that estimates the estimand, and an estimate is a result from the estimator. The estimand framework introduces 5 attributes for an estimand. They are treatments, variables, target populations, population-level summaries, and intercurrent events [4]. The 5 attributes together define an estimand. Generally, treatments are drugs used in clinical trials. They can be new drugs or new combinations of drugs. Variables are outcomes used to assess efficacy and safety of treatments, such as blood pressure and the occurrence rate of adverse events. They are also called endpoints. A target population is a group of people that satisfy specific conditions of clinical interest, such as people older than 60 years with hypertension. A population-level summary is a statistical approach to compare the treatment effect among different groups, such as the risk difference between the treatment arm and the placebo arm. Intercurrent events are events “occurring after treatment initiation that affect either the interpretation or the existence of the measurements associated with the clinical question of interest” [4]. Usually, they affect the definitions of treatments, variables, and target populations. Intercurrent events are very common in practice, including use of concomitant therapies, treatment switch, and death before endpoint measurement [4]. For example, when concomitant therapies are used, their effects are mixed with the treatment effect of interest, which may bias estimation of the treatment effect. When a participant in the treatment arm switches to the placebo arm, the drug effect on this participant no longer comes from the original treatment. When a participant dies before an endpoint assessment, the endpoint will become missing.

The estimand framework proposes 5 strategies to handle intercurrent events, with consideration of study objectives [4]. They are the treatment policy strategy, the hypothetical strategy, the composite variable strategy, the while on treatment strategy, and the principal stratum strategy [4]. The treatment policy strategy considers intercurrent events as part of the treatments being compared in clinical trials. The hypothetical strategy hypothesizes what would happen if no intercurrent event occurred. The composite variable strategy considers intercurrent events as part of the variables being assessed in clinical trials. The while on treatment strategy excludes any data after intercurrent events, including treatments and endpoints after intercurrent events. The principal stratum strategy considers the treatment effect in specific subpopulations of the entire target population. To illustrate the use of estimands in the real world, I have described estimands from some recent clinical trials in Multimedia Appendix 2 [43-48].

## A Causal Inference Framework Compared to Estimands

I would like to introduce a causal inference framework based on potential outcomes I have previously reported [11]. The framework described here is simplified: noncompliance and many important assumptions are not mentioned; only two treatment conditions are considered. However, a simplified framework is good for a broad audience when the definition of a treatment effect is accurate.

**Figure 1.**
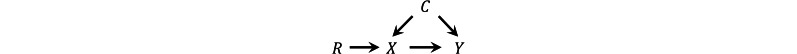
Causal directed acyclic graph for the treatment *X*, the endpoint *Y*, the randomization scheme *R*, and the confounders *C*.

Suppose there is a 2-arm randomized controlled clinical trial, with full compliance to treatment. This clinical trial has a sample size of N, a binary treatment X, a continuous endpoint Y, a randomization scheme R, and confounders C. R only affects X. X only affects Y. Hence, R affects Y only through X. C affects both X and Y. Their causal relationships can be shown in a causal directed acyclic graph (Figure 1).

 X, Y, R are random vectors of length N. C includes both measured and unmeasured confounders. It is a random matrix of row dimension N. $X_{i}$, $Y_{i}$, $R_{i}$, $C_{i}$ represent random variables or vectors for the participant i, where $\begin{array}{l} i\in \left\{1,2,3,\ldots ,N\right\} \end{array}$. $R_{i}=0$ means that the participant is assigned to the control arm, and $R_{i}=1$ means that the participant is assigned to the treatment arm. $\begin{array}{l} X_{i}\left(R_{i}=0\right)=0 \end{array}$ means that the participant takes the control treatment if assigned to the control arm, and $\begin{array}{l} X_{i}\left(R_{i}=1\right)=1 \end{array}$ means that the participant takes the experimental treatment that is of primary clinical interest if assigned to the treatment arm. $\begin{array}{l} Y_{i}\left(X_{i}\left(R_{i}=0\right)=0\right) \end{array}$ is the endpoint if the participant takes the control treatment as assigned, and $\begin{array}{l} Y_{i}\left(X_{i}\left(R_{i}=1\right)=1\right) \end{array}$ is the endpoint if the participant takes the experimental treatment as assigned. $R_{i}$, $X_{i}$ and $Y_{i}$ are potential outcomes.

The clinical goal is to estimate the overall treatment effect on the endpoint of taking the experimental treatment versus taking the control treatment among all participants. For the participant i, if they have $\begin{array}{l} X_{i}\left(R_{i}=0\right)=0 \end{array}$, then they have $\begin{array}{l} Y_{i}\left(X_{i}\left(R_{i}=0\right)=0\right) \end{array}$, and if they have $\begin{array}{l} X_{i}\left(R_{i}=1\right)=1 \end{array}$ , then they have $\begin{array}{l} Y_{i}\left(X_{i}\left(R_{i}=1\right)=1\right) \end{array}$. For this participant, the individual treatment effect (ITE) is defined as the difference between two potential outcomes of $Y_{i}$. That is,


$$ITE=Y_{i}\left(X_{i}\left(R_{i}=1\right)=1\right)-\;Y_{i}\left(X_{i}\left(R_{i}=0\right)=0\right).$$

 I further assume that the distribution of Y has linear functional forms with X and C. I also assume that the ITE is same for all participants. These assumptions can be relaxed theoretically [11]. The distribution of Y is thus assumed to be


$$Y=\beta_{0}+\beta_{1}X+\beta_{2}C+\varepsilon .$$

 Then, for the participant i,


$$Y_{i}(X_{i}(R_{i}=1)=1)=\beta_{0}+\beta_{1}+\beta_{2}C_{i}+\varepsilon_{i},$$

$$Y_{i}\left(X_{i}\left(R_{i}=0\right)=0\right)=\beta_{0}+\beta_{2}C_{i}+\varepsilon_{i}.$$

 Hence, $\begin{array}{l} \beta_{1}=Y_{i}\left(X_{i}\left(R_{i}=1\right)=1\right)-\;Y_{i}\left(X_{i}\left(R_{i}=0\right)=0\right) \end{array}$, and it equals the ITE for the participant i. The ITE indicates how the endpoint would change when only the treatment condition changes for this participant. There are N ITEs. Suppose a researcher is interested in an average treatment effect (ATE) of taking the experimental treatment versus taking the control treatment among all participants, which is the answer to the clinical goal mentioned above. The ATE is an average of all ITEs. It is defined as


$$\begin{array}{l} ATE=E\left(Y\left(X\left(R=1\right)=1\right)-Y\left(X\left(R=0\right)=0\right)\right) \end{array}.$$

This indicates that $\beta_{1}$ also equals the ATE, because $\begin{array}{l} ATE=E\left(\beta_{1}\right)=\beta_{1} \end{array}$. Under this specific distributional assumption of Y, $\beta_{1}$ acts as an estimator to the ATE. If the researcher makes different distributional assumptions, they will obtain different estimators.

Further, based on expectation properties, it is clear that


ATE=E(Y(X(R=1)=1))−E(Y(X(R=0)=0)).

 This also means that the estimand is the difference between the average outcome of taking the experimental treatment among all participants and the average outcome of taking the control treatment among all participants. The problem is that, in the real world, each participant only takes one kind of treatment, and only one of the two hypothetical situations with regard to two arms can happen. In this clinical trial setting, for each participant, the observed random assignment $R_{i}^{o}$ is either 0 or 1, the observed treatment $X_{i}^{o}$ is either $\begin{array}{l} X_{i}\left(R_{i}=0\right) \end{array}$ or $\begin{array}{l} X_{i}\left(R_{i}=1\right) \end{array}$, and the observed outcome $Y_{i}^{o}$ is either $\begin{array}{l} Y_{i}\left(X_{i}\left(R_{i}=0\right)\right) \end{array}$ or $\begin{array}{l} Y_{i}\left(X_{i}\left(R_{i}=1\right)\right) \end{array}$. The relationships between potential outcomes and observed variables can be described as


$$X_{i}^{o}=X_{i}(R_{i}=R_{i}^{o}),$$

$$Y_{i}^{o}=Y_{i}(X_{i}(R_{i}=R_{i}^{o})),$$

which implies that $Y^{o}=Y(X^{o})$. Through $Y^{o}=Y(X^{o})$ and the distribution of Y, the researcher has a causal linear model of observed variables to estimate the estimand. The linear model is given as


$$Y^{o}=\beta_{0}+\beta_{1}X^{o}+\beta_{2}C+\varepsilon ,$$

$$\begin{array}{l} E\left(\varepsilon \right)=0,\;Var\left(\varepsilon \right)=\sigma^{2}. \end{array}$$

 The linear model is the statistical model that the researcher builds using actual clinical trial data, where $\beta_{1}$ is not changed. After the linear model is built with data, an estimate ${\hat{\beta }}_{1}$ on $\beta_{1}$ would be obtained. ${\hat{\beta }}_{1}$ is an estimate of the ATE.

The statistical formula of the ATE as $\begin{array}{l} E\left(Y\left(X\left(R=1\right)=1\right)-Y\left(X\left(R=0\right)=0\right)\right) \end{array}$ already contains 4 attributes for an estimand, but it omits the attribute of intercurrent events. The attribute of treatments is represented by X. The attribute of variables is represented by Y. The attribute of population-level summaries is represented by the difference in the ATE between taking the experimental treatment and taking the control treatment among all participants, that is, the expectation form. The attribute of target population is implicitly stated as the clinical trial population and can be made explicit in the definition of the estimand. If a selection variable S indicates how the target population is selected from the general public, the formula can be updated to $\begin{array}{l} E\left(Y\left(X\left(R=1\right)=1\right)-Y\left(X\left(R=0\right)=0\right)|S\right) \end{array}$.

On the other hand, the 4 attributes of treatments, variables, population-level summaries, and target populations are not sufficient to define a clear treatment effect without the statistical formula for the ATE. The estimand framework raises awareness of a genuine treatment effect and makes progress on estimand attributes related to a treatment effect. If it can be improved with a clear statistical definition, it will gain a stronger theoretical foundation.

Let us take a look at the ITT principle. Under the ITT principle, a linear model of $Y^{o}$ that does not consider auxiliary variables such as measured variables in C could be


$$Y^{o}=\beta_{0}+\beta_{1}R^{o}+\varepsilon ,$$

$$\begin{array}{l} E\left(\varepsilon \right)=0,\;Var\left(\varepsilon \right)=\sigma^{2}, \end{array}$$

where $\beta_{1}$ now is an estimator of the average effect of the random assignment, or in other words, the average randomization effect (ARE). Note that not including auxiliary variables does not make this model invalid, but including auxiliary variables may improve model efficiency. The statistical formula for the ARE is $\begin{array}{l} E\left(Y\left(R=1\right)-Y\left(R=0\right)\right) \end{array}$, from which the ARE is the difference between the average outcome of being randomized to the treatment arm among all participants and the average outcome of being randomized to the control arm among all participants. Hence, the ARE is different from the ATE. The ARE does not represent a genuine effect of the experimental treatment compared to the control treatment. It is the randomization effect, or specifically, the effect of being randomized to an experimental arm versus being randomized to a control arm. When the estimand framework is used with the ITT principle, usually the researcher will obtain an estimate for the ARE rather than the ATE. It also indicates that the estimand framework may depend on the statistical analysis approach to define a “treatment” effect.

## Impact of Intercurrent Event Strategies on Treatment Effects

I would like to summarize how intercurrent events and their strategies are related to the ATE. I provide details for each intercurrent event strategy in Multimedia Appendix 3.

The treatment policy strategy includes intercurrent events in the definition of treatments. It defines a new treatment and a new endpoint. The new treatment is a combination of the original treatment and other treatments, such as the original treatment plus rescue therapy. It is also called a treatment policy [4]. The endpoint is affected by the new treatment, so it is different from the original endpoint. The ATE is changed to a genuine treatment effect at the endpoint of the experimental treatment policy versus the control treatment policy.

The hypothetical strategy hypothesizes nonexistence of intercurrent events and makes relevant data missing. Data missingness can be applied to the variable or the treatment, but it does not change the definition of the treatment and the variable, which means that the ATE will not be changed. Hence, this strategy can maintain the original treatment effect. It is more like a statistical imputation approach because suitable statistical methods should be used to impute missing data.

The composite variable strategy includes intercurrent events in the definition of endpoints. It defines a new composite endpoint that consists of both the original endpoint and a new component. For example, death could be a new component for an endpoint that measures serious adverse events [48]. Usually, the composite endpoint is broader than the original endpoint, and it is necessary to explain the rationale for the new component. The ATE is changed to a genuine treatment effect on the composite endpoint of the experimental treatment versus the control treatment.

The while on treatment strategy uses available data measured before intercurrent events and discards any data after the events. It defines a new treatment and a new endpoint by modifying the observation time of the original treatment and endpoint [4]. Intercurrent events usually make the observation time shorter than the planned duration, and the new treatment and endpoint last up to the occurrence of intercurrent events. The ATE is changed to a genuine treatment effect of the experimental treatment versus the control treatment before intercurrent events occur.

The principal stratum strategy estimates the ATE in a subpopulation of participants who would experience intercurrent events or a subpopulation who would not experience intercurrent events, instead of the original target population. Each subpopulation is a principal stratum [4]. For each participant, the treatment and the variable are not changed. That is, the ITE for each participant is not changed. The ATE becomes an average of all ITEs in the principal stratum. Hence, the ATE could be changed, especially under an assumption that the ITE is not identical for all participants.

**Figure 2.**
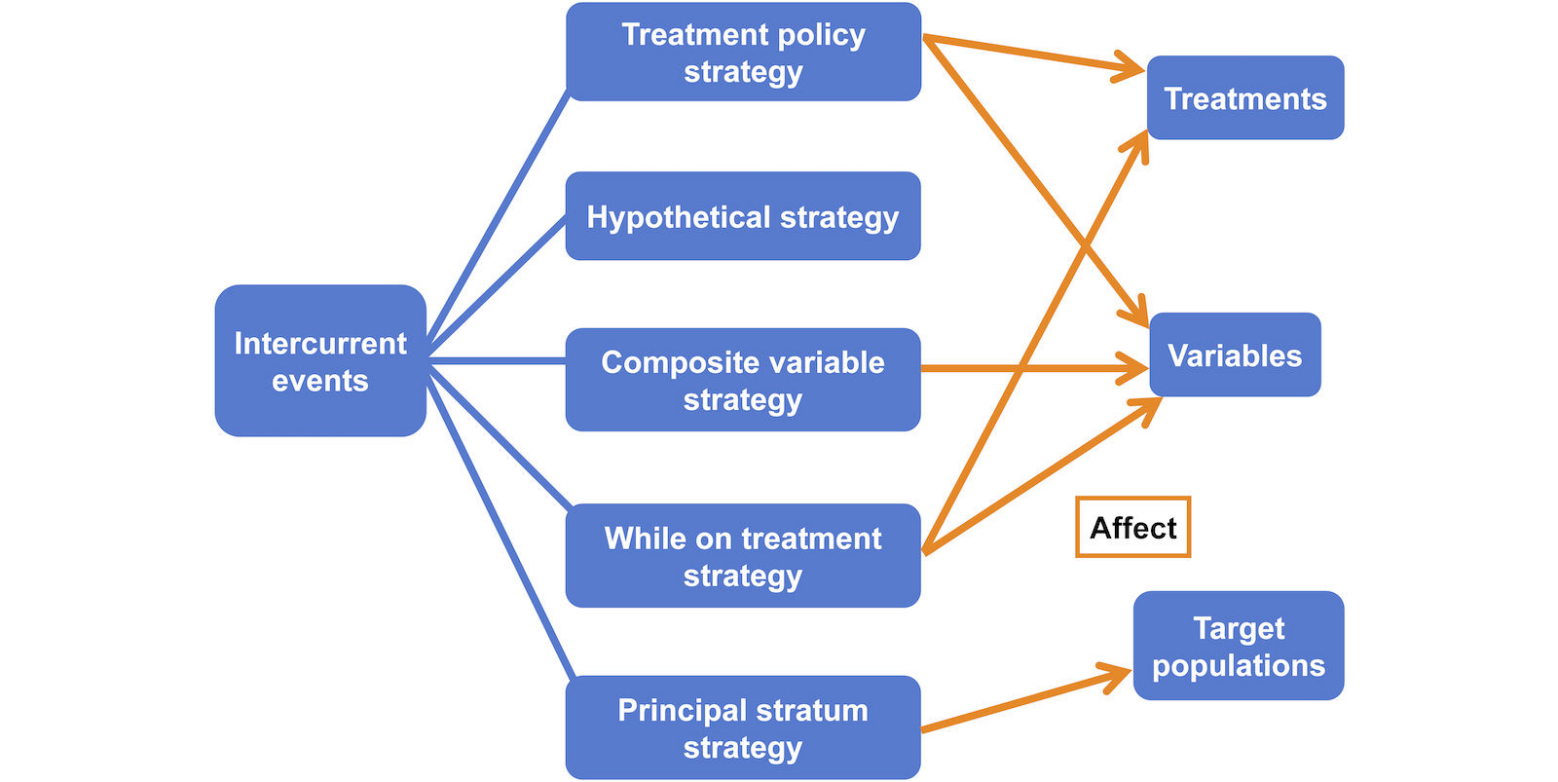
Different ways intercurrent event strategies control the impact of intercurrent events.

Intercurrent events indirectly affect the ATE by directly affecting the treatments, the variables, the target populations, or some of these estimand attributes at the same time. Intercurrent event strategies can control the impact of intercurrent events. They can direct the impact toward certain estimand attributes, or even stop the impact, as shown in Figure 2. For example, suppose an intercurrent event is discontinuation of trial treatment and it affects both the treatment and the variable [47]. The treatment policy strategy includes treatment discontinuation in the treatment policy and changes the treatment and the variable, while the hypothetical strategy makes data after treatment discontinuation missing and stops the impact of treatment discontinuation. Once we know how these strategies work, any further strategy could be interpreted similarly with regards to the treatments, the variables, and the target populations.

## Recommendations and Future Outlook

The estimand framework serves one more pragmatic purpose: it informs industry nonstatisticians of a precise definition of a treatment effect [8]. This practical value is highly appreciated, but we need to ask, “How can precision be guaranteed?” A theoretical foundation will not only guarantee a precise definition of treatment effects, but it will also guarantee a precise understanding of treatment effects. For many years, regulatory authorities and industry professionals have made great efforts to ensure good practice related to the estimand framework. However, this does not mean that every professional knows exactly what the estimand framework is doing. If professionals could be educated with an introduction to causal inference so that they understand what a treatment effect is, how intercurrent events affect it, and the impact of various intercurrent event strategies on treatment effects, it might be possible to establish an industry consensus on a precise understanding of treatment effects. This would make communication between statisticians and nonstatisticians less time-consuming, and planning and analysis of clinical trials would improve.

**Figure 3.**
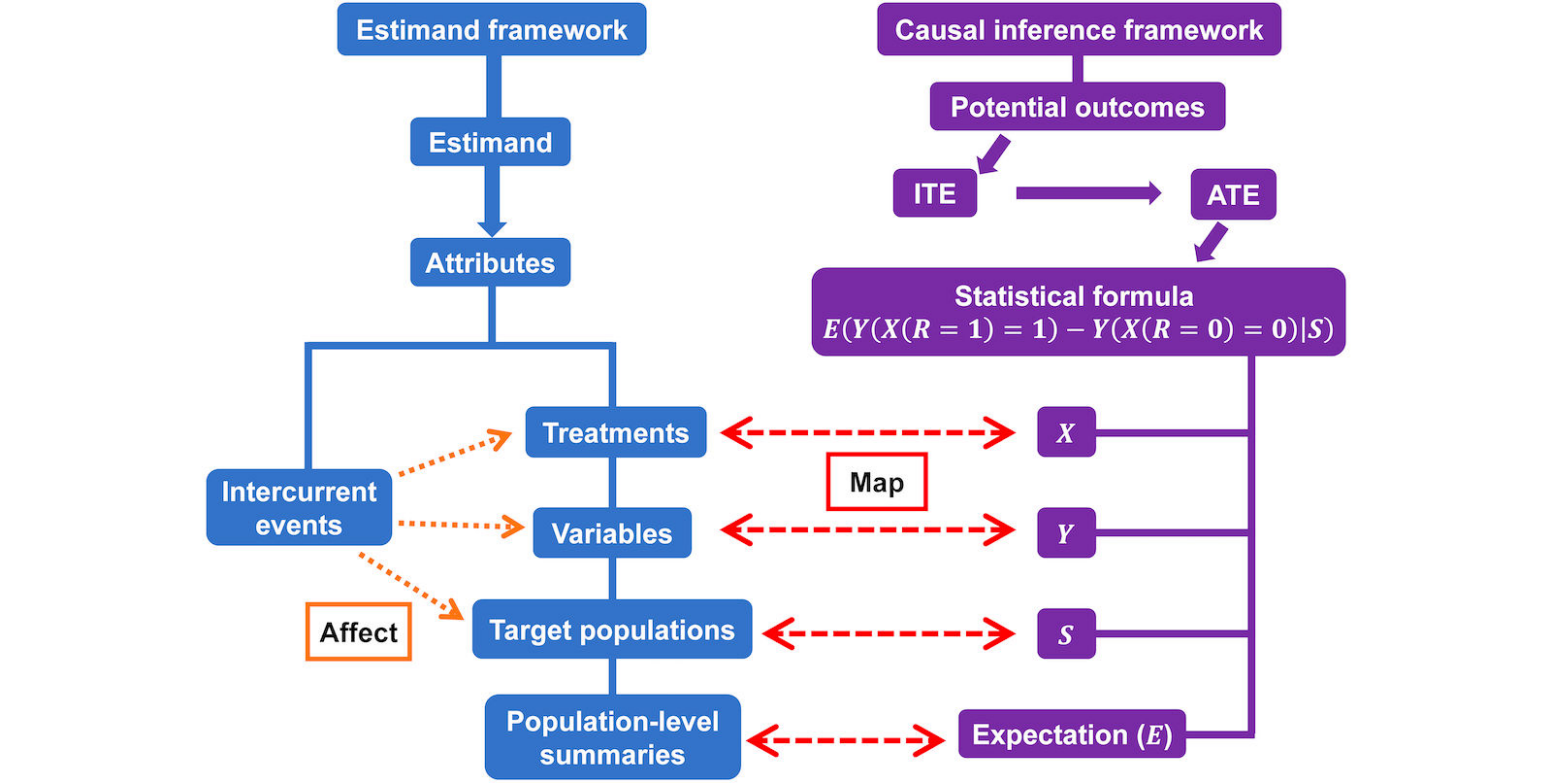
The attribute mapping process to connect the estimand framework and the causal inference framework. ATE: average treatment effect; ITE: individual treatment effect.

Hence, it will be useful to incorporate a causal inference framework into the estimand framework. The first question is, “Which causal inference framework should professionals use?” The potential outcome framework is a good start. It provides a clear definition of a genuine treatment effect. Some people may argue that choosing a causal inference framework is more related to data analysis. From the causal inference framework, we can see that $\beta_{1}$ in the linear model actually comes from construction of distributions for potential outcomes. A causal inference framework is a basis for subsequent statistical models, including linear models and more complex modeling approaches. Next, how could the causal inference framework be used in the estimand framework? I provide a novel way to map estimand attributes onto the statistical presentation of the ATE, and intercurrent events act as mediation mechanisms in the attribute mapping process, as shown in Figure 3. The treatment, the endpoint, the population and the expectation form from the statistical presentation of the ATE correspond in order to the treatment, the variable, the target population and the population-level summary from the estimand attributes. Intercurrent events themselves are not part of the statistical presentation of the ATE, but they modify the statistical presentation through treatments, variables and target populations. It implies that the ATE is not equivalent to the estimand, but the two concepts share core attributes, which provides a link between the estimand framework and the causal inference framework. Similar to Drury et al [8], I argue that the estimand framework and the causal inference framework are not “competing.” In addition, this link also indicates a possibility that the estimand framework may borrow theoretical benefits from the causal inference framework.

Interpretation of the estimand framework from a causal inference perspective is useful for clinical trials both in the pharmaceutical industry and in academia. When more and more academic clinical trials conduct analyses using the standardized estimand framework, communication about treatment effect results between the industry and academia may be greatly facilitated, which could improve the efficiency of clinical trials in a broad sense.

Further work is needed. For example, Figure 3 might have to be transformed into a format that professionals prefer. More complex situations, such as unmeasured confounding, noncompliance, and multiple treatment conditions, should be discussed. The causal inference framework discussed above can be expanded to deal with these issues. In addition to risk difference, more population-level summaries should also be discussed, such as relative risk and odds ratio. A review of statistical principles and models might be necessary to understand analytical performance under the estimand framework updated with causal inference theories.

## Supplementary material

10.2196/88813 Multimedia Appendix 1 A glossary of estimand-related concepts from E9 (R1).

10.2196/88813 Multimedia Appendix 2 Examples of estimands from recent clinical trials.

10.2196/88813 Multimedia Appendix 3 Impact of intercurrent event strategies on treatment effects.

## Abbreviations

ARE: average randomization effect
ATE: average treatment effect
ICH: International Council for Harmonisation of Technical Requirements for Pharmaceuticals for Human Use
ITE: individual treatment effect
ITT: intention-to-treat
