# Supplementary material for: Interpreting the Estimand Framework From a Causal Inference Perspective
Source: JMIRx Med. 2026 May 22;7:e88813. doi: 10.2196/88813 (PMC13202416; doi:10.2196/88813)
Supplement: Multimedia Appendix 1 [file xmed-v7-e88813-s001.pdf]

A glossary of estimand-related concepts from E9(R1) [1]

Concept	Definition
Estimand	A precise description of the treatment effect reflecting the clinical question posed by the trial objective. It summarises at a population-level what the outcomes would be in the same patients under different treatment conditions being compared.
Estimate	A numerical value computed by an estimator.
Estimator	A method of analysis to compute an estimate of the estimand using clinical trial data.
Treatment	The treatment condition of interest and, as appropriate, the alternative treatment condition to which comparison will be made. These might be individual interventions, combinations of interventions administered concurrently, eg, as add-on to standard of care, or might consist of an overall regimen involving a complex sequence of interventions.
Variable	The variable (or endpoint) to be obtained for each patient that is required to address the clinical question. The specification of the variable might include whether the patient experiences an intercurrent event.
Target population	The population of patients targeted by the clinical question. This will be represented by the entire trial population, a subgroup defined by a particular characteristic measured at baseline, or a principal stratum defined by the occurrence (or non-occurrence, depending on context) of a specific intercurrent event.
Population-level summary	A population-level summary for the variable provides a basis for comparison between treatment conditions.
Intercurrent events	Events occurring after treatment initiation that affect either the interpretation or the existence of the measurements associated with the clinical question of interest.
Treatment policy strategy	The occurrence of the intercurrent event is considered irrelevant in defining the treatment effect of interest: the value for the variable of interest is used regardless of whether or not the intercurrent event occurs.
Hypothetical strategy	A scenario is envisaged in which the intercurrent event would not occur: the value of the variable to reflect the clinical question of interest is the value which the variable would have taken in the hypothetical scenario defined.
Composite variable strategy	An intercurrent event is considered in itself to be informative about the patient's outcome and is therefore incorporated into the definition of the variable.
While on treatment strategy	Response to treatment prior to the occurrence of the intercurrent event is of interest. Terminology for this strategy will depend on the intercurrent event of interest, eg, "while alive", when considering death as an intercurrent event.
Principal stratum strategy	The target population might be taken to be the "principal stratum" in which an intercurrent event would occur. Alternatively, the target population might be taken to be the principal stratum in which an intercurrent event would not occur. The clinical question of interest relates to the treatment effect only within the principal stratum.
Principal stratification	Classification of subjects according to the potential occurrence of an intercurrent event on all treatments. With two treatments, there are four principal strata with respect to a given intercurrent event: subjects who would not experience the event on either treatment, subjects who would experience the event on treatment A but not B, subjects who would experience the event

	on treatment B but not A, and subjects who would experience the event on both treatments.
Principal stratum	Any of the strata (or combination of strata) defined by principal stratification.

References

1. International Council for Harmonisation of Technical Requirements for Pharmaceuticals for Human Use. E9(R1)-Addendum on estimands and sensitivity analysis in clinical trials to the guideline of statistical principles for clinical trials. 2019. www.ich.org/page/efficacy-guidelines#9-2 [accessed Oct 30, 2023].
