# Supplementary material for: Interpreting the Estimand Framework From a Causal Inference Perspective
Source: JMIRx Med. 2026 May 22;7:e88813. doi: 10.2196/88813 (PMC13202416; doi:10.2196/88813)
Supplement: Multimedia Appendix 2 [file xmed-v7-e88813-s002.pdf]

Examples of estimands from recent clinical trials

Clinical Trial	#	Estimand
**Title**: A randomized, parallel-arm, double-blind study of efficacy and safety of dulaglutide when added to SGLT2 inhibitors in patients with type 2 diabetes mellitus [1]<br>**Sponsor**: Eli Lilly and Company<br>**Phase**: 3	1	**Treatment**: Dulaglutide and placebo, added to SGLT2 inhibitors<br>**Variable**: Change from baseline in hemoglobin A1c at week 24<br>**Target population**: Patients with inadequately controlled with type 2 diabetes mellitus on concomitant SGLT2 inhibitor therapy<br>**Population-level summary**: Difference in the variable between groups<br>**Intercurrent event**: Use of glucose-lowering rescue therapy after randomization<br>• While on treatment strategy with the ITT principle, excluding post-rescue data<br>• Treatment policy strategy with the ITT principle, including post-rescue data
**Title**: Effect and safety of liraglutide 3.0 mg as an adjunct to intensive behavior therapy for obesity in a non-specialist setting (SCALE IBT) [2]<br>**Sponsor**: Novo Nordisk A/S<br>Phase: 3<br>**Type**: Randomized, double-blind, placebo controlled, two-armed, multicenter	2	**Treatment**: liraglutide and placebo, as an adjunct to intensive behavior therapy<br>**Variable**: Proportion of participants losing at least 5% of baseline body weight at week 56<br>**Target populati**on: People with obesity and without type 1 or 2 diabetes mellitus<br>**Population-level summary**: Odds ratio between groups<br>**Intercurrent event**: Nonadherence to treatment<br>• Treatment policy strategy, regardless of adherence to treatment
**Title**: A two-part seamless, multi-center randomized, placebo-controlled, double-blind study to investigate the safety, tolerability, pharmacokinetics, pharmacodynamics and efficacy of risdiplam (RO7034067) in type 2 and 3 spinal muscular atrophy patients [3]<br>**Sponsor**: Hoffmann-La Roche<br>**Phase**: 2	3	**Treatment**: Risdiplam and placebo<br>**Variable**: Change in the Total Motor Function Measure 32 total score from baseline to month 12<br>**Target population**: Type 2 and non-ambulant type 3 spinal muscular atrophy patients aged 2-25<br>**Population-level summary**: Difference in the variable between groups.<br>**Intercurrent event**: Use of prohibited medications to treat spinal muscular atrophy, discontinuation of trial treatment<br>• Hypothetical strategy, envisaging no intercurrent event occurs
**Title**: A randomized, open-label, cross-over, placebo inhaler study to evaluate the correct use of ELLIPTA dry powder inhaler (DPI) compared to DISKUS DPI used in combination with HandiHaler DPI in participants with chronic obstructive pulmonary disease (COPD) [4]<br>**Sponsor**: GlaxoSmithKline<br>**Phase**: 4	4	**Treatment**: ELLIPTA placebo DPI, DISKUS placebo DPI, HandiHaler placebo DPI<br>**Variable**: Percentage of participants with zero errors after 28 days of inhaler use<br>**Target population**: Patients with COPD, aged no less than 40<br>**Population-level summary**: Odds ratio between groups<br>**Intercurrent event**: Withdrawal from the study, attending visits without the device(s) participants are randomized to<br>• Hypothetical strategy
**Title**: Efficacy and safety of the SQ tree sublingual immunotherapy tablet in children and adolescents (5 through 17 years of age) with	5	**Treatment**: SQ tree sublingual immunotherapy tablet and placebo, with rescue medication taken<br>**Variable**: Average total combined score during the birch pollen season

moderate to severe allergic rhinitis and/or conjunctivitis induced by pollen from birch and trees belonging to the birch homologous group [5]<br>**Sponsor**: ALK-Abelló A/S<br>**Phase**: 3<br>**Type**: Randomized, parallel-group, double-blind, placebo-controlled		**Target population**: Children and adolescents aged 5-17 with moderate to severe allergic rhinitis and/or conjunctivitis induced by pollen from birch<br>**Population-level summary**: Difference in the variable between groups<br>**Intercurrent event**: Discontinuation of trial treatment<br>• Hypothetical strategy<br>• Treatment policy strategy
**Title**: A phase 2, randomized, double-blind, placebo-controlled trial to evaluate safety, tolerability, and immune responses of an investigational monovalent chimpanzee adenoviral-vectored Marburg virus vaccine in healthy adults [6]<br>**Sponsor**: Albert B. Sabin Vaccine Institute	6	**Treatment**: cAd3-Marburg vaccine and placebo<br>**Variable**: Percentage of vaccinated participants who would develop serious adverse events<br>**Target population**: Healthy adults aged 18- 70<br>**Population-level summary**[a]: Odds ratio between groups<br>**Intercurrent event**: Infection or death related to adverse events during the study<br>• Composite variable strategy, treating the intercurrent event as serious adverse events
	7	**Treatment**: cAd3-Marburg vaccine and placebo<br>**Variable**: Geometric mean concentration of anti-Marburg-GP binding IgG antibodies at day 29 post-vaccination<br>**Target population**: Healthy adults aged 18-70<br>**Population-level summary**[a]: Difference in the variable between groups<br>**Intercurrent event 1**: Subsequent Marburg virus infection or influence from immune-modifying drugs or non-study vaccines<br>• Hypothetical strategy<br>**Intercurrent event 2**: Current (or prior) Marburg virus infection at time of vaccination<br>• Principal stratum strategy, excluding participants with active or prior Marburg virus infection at the time of vaccination

[a] The population-level summary is defined by the author, as it has not been officially published.

References

1. Eli Lilly and Company. A study of dulaglutide (LY2189265) in participants with type 2 diabetes mellitus (AWARD-10). ClinicalTrials.gov. 2015. https://clinicaltrials.gov/study/NCT02597049 [accessed Feb 20, 2026].

2. Novo Nordisk A/S. Effect and safety of liraglutide 3.0 mg as an adjunct to intensive behavior therapy for obesity in a non-specialist setting (SCALE IBT). ClinicalTrials.gov. 2016. https://clinicaltrials.gov/study/NCT02963935 [accessed Feb 20, 2026].

3. Hoffmann-La Roche. A study to investigate the safety, tolerability, pharmacokinetics, pharmacodynamics and efficacy of risdiplam (RO7034067) in type 2 and 3 spinal muscular atrophy (SMA) participants (SUNFISH). ClinicalTrials.gov. 2016. https://clinicaltrials.gov/study/NCT02908685 [accessed Feb 20, 2026].

4. GlaxoSmithKline. Comparative study of ELLIPTA dry powder inhaler (DPI) versus DISKUS DPI used with HandiHaler DPI in subjects with chronic obstructive pulmonary disease (COPD). ClinicalTrials.gov. 2017. https://clinicaltrials.gov/study/NCT03227445 [accessed Feb 20, 2026].

5. ALK-Abelló A/S. A study in children and adolescents with birch pollen-induced rhinoconjunctivitis (TreeTop). ClinicalTrials.gov. 2021. https://clinicaltrials.gov/study/NCT04878354 [accessed Feb 20, 2026].

6. Albert B. Sabin Vaccine Institute. Monovalent chimpanzee adenoviral-vectored Marburg virus vaccine in healthy adults. ClinicalTrials.gov. 2023. https://clinicaltrials.gov/study/NCT05817422 [accessed Feb 20, 2026].
