# Supplementary material for: Interpreting the Estimand Framework From a Causal Inference Perspective
Source: JMIRx Med. 2026 May 22;7:e88813. doi: 10.2196/88813 (PMC13202416; doi:10.2196/88813)
Supplement: Multimedia Appendix 3 [file xmed-v7-e88813-s003.pdf]

The treatment policy strategy

The treatment policy strategy is to include intercurrent events in the definition of treatments. Under this strategy, "the value for the variable of interest is used regardless of whether or not the intercurrent event occurs" [1]. Considering Estimand 1 in Multimedia Appendix 2, when participants use glucose-lowering rescue therapy after treatment initiation, the hemoglobin A1c level will be affected by rescue therapy in addition to dulaglutide or placebo. In this case, people cannot estimate the ATE of dulaglutide versus placebo on hemoglobin A1c without adjusting for rescue therapy. The treatment policy strategy will regard rescue therapy as part of treatment, and compare a treatment policy of dulaglutide plus rescue therapy added to SGLT2 inhibitors with a treatment policy of placebo plus rescue therapy added to SGLT2 inhibitors. Sometimes, use of rescue therapy is common in clinical practice due to ethical or other reasons, which makes a treatment policy acceptable. Considering Estimand 2 in Multimedia Appendix 2, when participants randomized to liraglutide do not adhere to the planned treatment plan, for example, they do not take liraglutide for a long period after treatment initiation, their liraglutide treatment intensity will become lower than that of participants who take liraglutide as planned. In this case, the actual effect of liraglutide as an adjunct to intensive behavior therapy on body weight may be reduced. The treatment policy strategy will ignore nonadherece to treatment and compare a treatment policy of liraglutide as an adjunct to intensive behavior therapy regardless of adherence with a treatment policy of placebo as an adjunct to intensive behavior therapy regardless of adherence.

From a statistical perspective, the treatment policy strategy defines a new treatment denoted by X' and a new endpoint denoted by Y', and compare different treatment policies. For the participant i, $X_i'(R_i = 0) = 0$ means that the participant would take the control treatment policy if assigned to the control arm, and $X_i'(R_i = 1) = 1$ means that the participant would take the experimental treatment policy if assigned to the treatment arm. For example, the control treatment policy is placebo plus rescue therapy added to SGLT2 inhibitors, and the experimental treatment policy is dulaglutide plus rescue therapy added to SGLT2 inhibitors. $Y_i'(X_i'(R_i = 0) = 0)$ would be the endpoint if the participant took the control treatment policy as assigned, and $Y_i'(X_i'(R_i = 1) = 1)$ would be the endpoint if the participant took the experimental treatment policy as assigned. Now the statistical formula of the ATE becomes $E(Y'(X'(R = 1) = 1) - Y'(X'(R = 0) = 0))$, different from the original ATE as $E(Y(X(R = 1) = 1) - Y(X(R = 0) = 0))$. The new statistical formula of the ATE represents a genuine average treatment effect on the endpoint of the experimental treatment policy versus the control treatment policy. Under the ITT principle, the randomization scheme will not be changed by the treatment policy strategy, but the statistical formula of the ARE becomes $E\big(Y'(R = 1) - Y'(R = 0)\big)$, also different from the original ARE as $E\big(Y(R = 1) - Y(R = 0)\big)$. The new statistical formula of the ARE represents a genuine average effect on the new endpoint of being assigned to the experimental arm versus being assigned to the control arm.

The hypothetical strategy

The hypothetical strategy is to hypothesize non-existence of intercurrent events and make relevant data missing. Under this strategy, "the value of the variable to reflect the clinical question of interest is the value which the variable would have taken in the hypothetical scenario defined" [1]. Often, the variable is hypothesized, and suitable statistical methods should be used to impute missing endpoints. Considering Estimand 3 in Multimedia Appendix 2, when participants use prohibited medications to treat spinal muscular atrophy after treatment initiation, the motor function measure may be affected by prohibited medications. If prohibited medications are effective, subsequent functional measurements will be improved and thus the apparent effect of risdiplam or placebo will become larger. In this case, people cannot directly use subsequent functional measurements after use of prohibited medications in statistical analysis. The hypothetical strategy will exclude subsequent functional measurements after use of prohibited medications, making the values of the variable missing after use of prohibited medications, and then use the remaining data to impute these missing values of the variable. The imputed values are what people would get if prohibited medications were not used. Considering Estimand 4 in Multimedia Appendix 2, when participants attend visits without the device(s) they are randomized to, correct use of their device(s) cannot be assessed and thus the outcome of how many errors participants make during use of the device(s) is naturally missing. In this case, the hypothetical strategy does not need to make data missing. It just needs to impute these missing outcomes as if participants brought their device(s) to visits.

In the above two examples, neither the definition of the treatment nor the definition of the variable are changed by the hypothetical strategy. The statistical formula of the ATE is still $E(Y(X(R=1)=1)-Y(X(R=0)=0))$, which represnts the original ATE of the experimental treatment versus the control treatment. Hence, the hypothetical strategy is able to estimate a genuine treatment effect. It is more like a statistical imputation approach. However, clear descriptions of the hypothetical scenario is required to justify use of this strategy [1].

Further, the hypothetical strategy can deal with situations beyond missingness only in the variable, as in the above two examples. Considering Estimand 5 in Multimedia Appendix 2, the birch pollen season lasts approximately 3 to 4 weeks, when participants take SQ tree sublingual immunotherapy tablet and placebo daily. If participants discontinue trial treatment before average total combined score assessments, subsequent treatment information and average total combined score assessments will both become missing. In this case, there will be missing data in both the treatment and the variable for Estimand 5. The hypothetical strategy will hypothesize what if these participants continued treatment and took subsequent assessments as planned, and then it will impute missing data based on statistical models researchers choose. In this case, the definitions of the treatment and the variable as well as the statistical formula of the ATE are not changed. Since hypothetical scenarios discussed here are not exhaustive, it is recommended to see how the statistical formula of the ATE would be changed based on specific situations the hypothetical strategy is applied on.

The composite variable strategy

The composite variable strategy is to include intercurrent events in the definition of endpoints. Under this strategy, intercurrent events are "informative about the patient's outcome and is therefore incorporated into the definition of the variable" [1]. Considering Estimand 6 in Multimedia Appendix 2, the clinical trial is a vaccine trial for a prevention purpose, where researchers want to evaluate safety of cAd3-Marburg vaccine. One primary approach is to evaluate serious adverse events in vaccinated participants. Participants may get infected with Marburg virus or die after vaccination, which will affect subsequent observation of serious adverse events. When Marburg virus infection occurs, it will be hard to tell if subsequent serious adverse events are due to Marburg virus infection or trial treatment. When death occurs, adverse events or any other events cannot be observed any longer. Through the composite variable strategy, Marburg virus infection and death after vaccination are considered serious adverse events. Usually, whether infection is a serious adverse event should depend on the infection severity. However, the composite variable strategy treats Marburg virus infection as a serious adverse event, regardless of its severity. This is appropriate for a vaccine, because infection may indicate a failure of trial treatment in prevention against Marburg virus, which is a serious outcome for a vaccine. If death is related to adverse events, it is usually reported as a serious adverse event in clinical practice. Hence, the composite variable strategy is also appropriate for death. The treatment is not changed under this strategy, while the variable is changed from serious adverse events to serious adverse events with infection and death always included.

From a statistical perspective, the composite variable strategy defines a new composite endpoint denoted by Y' that consists of both the original endpoint Y and a new component that makes sense to Y. Usually, the composite endpoint is broader than the original endpoint, and it is necessary to explain the rationale for the new component. $Y_i'(X_i(R_i = 0) = 0)$ would be the composite endpoint if the participant took the control treatment as assigned, and $Y_i'(X_i(R_i = 1) = 1)$ would be the composite endpoint if the participant took the experimental treatment as assigned. Now the statistical formula of the ATE becomes $E(Y'(X(R = 1) = 1) - Y'(X(R = 0) = 0))$. The new statistical formula of the ATE represents a genuine ATE on the composite endpoint of the experimental treatment versus the control treatment. The statistical formula of the ARE becomes $E\big(Y'(R = 1) - Y'(R = 0)\big)$. The new statistical formula of the ARE represents a genuine ARE on the composite endpoint of being assigned to the experimental arm versus being assigned to the control arm.

The while on treatment strategy

The while on treatment strategy is to use available data measured before intercurrent events and discard any data afterwards. Under this strategy, "response to treatment prior to the occurrence of the intercurrent event is of interest" [1]. Considering Estimand 1 in Multimedia Appendix 2, participants use glucose-lowering rescue therapy after treatment initiation. Suppose people are not

interested in rescue therapy but in trial treatments only. People want to see how the level of hemoglobin A1c would be affected only by dulaglutide or placebo added to SGLT2 inhibitors. Through the while on treatment strategy, all data after use of rescue therapy will be excluded. Treatment information and hemoglobin A1c assessments after use of rescue therapy will not be used in statistical analysis.

From a statistical perspective, the while on treatment strategy defines a new treatment denoted by X' and a new endpoint denoted by Y'. Different from the treatment policy strategy, the while on treatment strategy modifies the observation time of the treatment and the endpoint [1]. When the intercurrent event is considered, the observation time may be shorter than the planned duration. For the participant i, $X_i'(R_i = 0) = 0$ means that the participant would take the control treatment before the intercurrent event occurs if assigned to the control arm, and $X_i'(R_i = 1) = 1$ means that the participant would take the experimental treatment before the intercurrent event occurs if assigned to the treatment arm. $Y_i'(X_i'(R_i = 0) = 0)$ would be the endpoint before the intercurrent event occurs if the participant took the control treatment as assigned, and $Y_i'(X_i'(R_i = 1) = 1)$ would be the endpoint before the intercurrent event occurs if the participant took the experimental treatment as assigned. Now the statistical formula of the ATE becomes $E(Y'(X'(R = 1) = 1) - Y'(X'(R = 0) = 0))$. The new statistical formula of the ATE represents a genuine ATE of the experimental treatment versus the control treatment before the intercurrent event occurs. The statistical formula of the ARE becomes $E\big(Y'(R = 1) - Y'(R = 0)\big)$. The new statistical formula of the ARE represents a genuine ARE on the endpoint of being assigned to the experimental arm versus being assigned to the control arm before the intercurrent event occurs.

For the same intercurrent event, multiple strategies may be applicable. Considering Estimand 5 in Multimedia Appendix 2, if participants discontinue trial treatment and then they stay in the study and finish average total combined score assessments, the treatment information will become missing but the endpoint assessment will not become missing after the intercurrent event occurs. The while on treatment strategy will exclude data of average total combined score assessments after discontinuation of trial treatment, while the hypothetical strategy may make average total combined score assessments after discontinuation of trial treatment missing and impute the missing assessments as if trial treatment was continued. The treatment policy strategy will regard discontinuation of trial treatment as part of a treatment policy, and it will use data of average total combined score assessments after discontinuation of trial treatment. However, the ATE is different among these different strategies, from discussions above. Choosing an appropriate intercurrent event strategy may need a clear statistical definition of the treatment effect and consideration on the clinical and/or regulatory interest [1].

The principal stratum strategy

The principal stratum strategy is to identify a subpopulation of participants who would or would not experience intercurrent events and estimate the ATE in this subpopulation instead of the entire

target population. This subpopulation is also called the principal stratum [1]. Please refer to Multimedia Appendix 1 for a definition. Under this strategy, “the clinical question of interest relates to the treatment effect only within the principal stratum” [1].

Let us hypothesize a randomized, placebo-controlled clinical trial with an experimental treatment taken mulitple times in a planned duration. Suppose now a potential interccurent event is that some participants would not tolerate treatment after treatment initiation and then they would discontinue trial treatment, which finally would change subsequent trial treatment and endpoint assessments. Unlike the actual intercurrent event where people actually see participants develop symptoms due to non-tolerance to treatment, the potential intercurrent event is a potential outcome where people imagine participants would not tolerate treatment. It is not yet observed, similar to potential outcomes in the causal inference framework [1]. The potential intercurrent event represents some instrinsic characteristic of participants, since tolerability to treatment toxicity is an intrinsic characteristic of humans. Suppose people are interested in a genuine treatment effect in participants who would tolerate treatment toxicity. Let tolerability be denoted by T. $T = 0$ represents not tolerating treatment toxicity, and $T = 1$ represents tolerating treatment toxicity. Then, four subpopulations can be defined and represented by P. $P = 1$ are participants who would tolerate both the control and experimental treatments if they took both treatments. $P = 2$ are participants who would tolerate neither the control treatment nor the experimental treatment if they took both treatments. $P = 3$ are participants who would tolerate the control treatment and would not tolerate the experimental treatment if they took both treatments. $P = 4$ are participants who would not tolerate the control treatment and would tolerate the experimental treatment if they took both treatments. People may be interested in the subpopulation of participants who would tolerate both treatments, that is $P = 1$, which would become the new target population. These four subpopulations are different from subgroups of participants who tolerate or do not tolerate treatment toxicity in the real world. In the above hypothesized trial, suppose people actually see a subgroup of participants in the control arm who tolerate the control treatment and complete trial treatment. From a potential outcome point of view, in this subgroup, some participants would tolerate the experimental treatment if assigned to the treatment arm instead, while the others would not tolerate the experimental treatment if assigned to the treatment arm instead. Hence, this subgroup may be a mix of $P = 1$ and $P = 3$.

Construction of the principal stratum requires careful justification. Not all subpopulations are appropriate in the clinical context. For example, in the above hypothesized trial, researchers may think it is unlikely that participants do not tolerate placebo and thus regard $P = 2$ and $P = 4$ as inappropriate strata. Considering Estimand 7 in Multimedia Appendix 2, participants may have already been infected with Marburg virus at time of vaccination. This intercurrent event is possible, because participants are exposed to Marburg virus in a time gap between enrollment to the trial and vaccination. From participants who have the intercurrent event, people cannot assess the preventional effect of cAd3-Marburg vaccine. People are more interested in the preventional effect of cAd3-Marburg vaccine on participants with no infection at time of vaccination. Since vaccination occurs after current or prior infection, current or prior infection is not related to vaccination from a

causal inference perspective. Two subpopulations can be defined and represented by P'. $P' = 1$ are participants who would not have current or prior Marburg virus at time of vaccination. $P' = 2$ are participants who would have current or prior Marburg virus at time of vaccination. Through the principal stratum strategy, $P' = 1$ will become the new target population. Other smaller strata, such as participants who would not have current or prior Marburg virus at time of vaccination and would get vaccinated later, or participants who would not have current or prior Marburg virus at time of vaccination and would not get vaccinated later, may not be necessary.

From a statistical perspective, the principal stratum strategy defines a principal stratum denoted by S' from the original target population S. For each participant, the treatment and the variable are not changed. Only the population of interest is changed [1]. Now the statistical formula of the ATE becomes $E(Y(X(R = 1) = 1) - Y(X(R = 0) = 0)|S')$. The new statistical formula of the ATE represents a genuine ATE in the principal stratum. As for the ITT principle, selection of the principal stratum could maintain randomization, if people do not exclude participants outside of the principal stratum from statistical analysis. For example, people may build statistical models on the ITT population and include an interaction term between strata P and randomization R, so that they can estimate the randomization effect in some specific stratum without breaking randomization. In this case, the statistical formula of the ARE becomes $E(Y(R = 1) - Y(R = 0)|S')$. The new statistical formula of the ARE represents a genuine ARE on the endpoint of being assigned to the experimental arm versus being assigned to the control arm in the principal stratum. It is recommended to consider how to use the principal stratum strategy with the ITT principle under specific clinical trial backgrounds.

References

1. International Council for Harmonisation of Technical Requirements for Pharmaceuticals for Human Use. E9(R1)-Addendum on estimands and sensitivity analysis in clinical trials to the guideline of statistical principles for clinical trials. 2019. www.ich.org/page/efficacy-guidelines#9-2 [accessed Oct 30, 2023].
